# Disclosing the Truth: A Dilemma between Instilling Hope and Respecting Patient Autonomy in Everyday Clinical Practice

**DOI:** 10.5539/gjhs.v6n1p128

**Published:** 2013-12-20

**Authors:** Pavlos Sarafis, Andreas Tsounis, Maria Malliarou, Eleni Lahana

**Affiliations:** 1 Faculty of Nursing, Technological Educational Institute, Lamia, Greece; 2 Centers for the Prevention of Addictions and Promoting Psychosocial Health of Municipality of Thessaloniki, Thessaloniki, Greece; 3 Faculty of Nursing, Technological Educational Institute, Larisa, Greece

**Keywords:** disclosure, truth, autonomy, cultural influences, ethics, hope, cancer

## Abstract

**Background:**

While medical ethics place a high value on providing truthful information to patients, disclosure practices are far from being the norm in many countries. Transmitting bad news still remains a big problem that health care professionals face in their every day clinical practice.

**Aims:**

Through the review of relevant literature, an attempt to examine the trends in this issue worldwide will be made.

**Method:**

Various electronic databases were searched by the authors and through systematic selection 51 scientific articles were identified that this literature review is based on.

**Results:**

There are many parameters that lead to the concealment of truth. Factors related to doctors, patients and their close environment, still maintain a strong resistance against disclosure of diagnosis and prognosis in terminally ill patients, while cultural influences lead to different approaches in various countries. Withholding the truth is mainly based in the fear of causing despair to patients. However, fostering a spurious hope, hides the danger of its’ total loss, while it can disturb patient-doctor relationship.

## 1. Introduction

Since the first half of twentieth century the dominant view about truth telling in terminally ill patients, was influenced by a paternalistic tradition. Doctors were recognized as the only decision makers, who could act on their own judgment, withholding information from patient, if they thought that this could cause harm (Tabak, Itzhaki, Sharon, & Barnoy, 2012).

In recent decades most physicians accept as ethical the proposition that patients are entitled to know the whole truth about their diagnosis (Freedman, 1993; Field, 1998).

However, since nowadays the issue of telling the patient the truth about their diagnosis and treatment options is approached differently in various countries, while there is still no global consensus on it (Georgaki, Kalaidopoulou, Liarmakopoulos, & Mystakidou, 2002; Tieying, Haishan, Meizhen, Yan, & Pengqian, 2011).

Most of the times truth telling means conveying bad news, like to confirm a serious cancer or a relapse of a disease, a metastasis or that a treatment was ineffective. That’s why the majority of doctors or nurses encounter many difficulties during truth telling, which lead them to feel psychological distress, impacting negatively on their emotional well-being and work satisfaction (Panagopoulou, Mintziori, Montgomery, Kapoukranidou, & Benos, 2008; Hulsman et al., 2010; Tang, Fang, Chun-Kai Fang, & Fujimori, 2013).

As a result, despite a global trend towards providing more information for patients, non-disclosure still dominates in every day clinical practice in various countries (Mystakidou, Parpa, Tsilila, Katsouda, & Vlahos, 2004; Surbone, 2004).

The aim of this article is to explore the reasons for attitudes of non-disclosure. As truth telling touches the core of medical ethics, we begin with the issue of patients’ autonomy. Then, the main factors that contribute to non-disclosure of diagnosis will be presented. Dilemmas and circumstances which correlate with there different perspectives (physicians’, patients’ and relatives’ perspective), as also other elements that increase difficulties, will be examined. Afterwards, the cultural context and the differences between various countries will be presented in a summary form. Finally, the main question that permeates through the whole study, which is associated with the fact of being or not the truth harmful, will be placed in the center of our interest.

## 2. Patient’s Autonomy

The overarching ethical principle which influenced modern medical thought during the 20^th^ century, became the respect for patient autonomy. The patients’ right to be fully informed of their medical condition, was based in the individual’ s right of freedom to make decisions on his/her own behalf, without undue manipulation by others (Gold, 2004).

Changes in doctor-patient relationship are impressed in a numerous of official declarations. According to the International Code of Medical Ethics, as it was amended by the 57^th^ WMAs’ General Assembly (2006), a physician shall act in the patient’ s best interest when providing medical care, respecting his/her right to accept or refuse treatment. At the same time, in the WMA’s Declaration on the Rights of the Patient, which was revised by its 171^st^ Council’s Session (2005), it is stressed that the patient has the right to self-determination, while the physician must inform him/her of the consequences of his/her decisions.

Besides, apart from declarations, in most western countries there is a legal requirement for doctors to inform patients of their condition, options and material risks of treatment. The only exception to this is in emergency situations, where a patient cannot provide consent and delay in treatment would result in death (Schwartz, Preece, & Hendry, 2002).

Meanwhile, the progressive availability of new treatments, especially as far as incurable diseases are concerned, has resulted in increased difficulty in evaluating the cost-benefit ratio for patient. It has also required patients to take on a greater role in the final decision-making. This means that patients need more information in order to participate substantially in this process (Gold, 2004).

At the same time, withholding the truth can damage the relationship between the doctor and the patient. Current researches suggest that in many cases patients interpret hidden information as health professionals withhold bad news (Fallowfield, Jenkins, & Beveridge, 2002; Tattersall, Butow, & Clayton, 2002).

Trust unsettling may also have a bad influence on patients’ adherence to therapy, which is crucial for its effectiveness (Kazdaglis et al., 2010).

However, according to many physicians, severe illness can compromise a patient’s capacity for autonomous decision-making, because the experience of pain, fear and anger, prevent them from “taking in” all the relevant information. Negative emotional state can impair their ability to make reasoned treatment choices with many different ways (Beste, 2005).

Besides, it is impossible for the patient to get a knowledge equivalent to that of a doctor, while in many cases this could be incomprehensible. At the same time leaving decision making entirely in the hands of the patient is a kind of an abdication of doctors’ responsibility (Gold, 2004).

Especially when many patients rely on the doctors view. Indeed, since nowadays there is a number of cancer patients who still want the doctor to have a dominant role in decision-making (Butow, Tattersall, & Goldstein, 1997).

Enhancing patients’ autonomy means helping them to make their own decisions and not forcing the truth on them, especially when they might not be prepared to deal with it. This makes balancing between autonomy and beneficence, which is already one of the most difficult ethical issues to navigate, even more complicated.

A summary of basic concepts about patient’s participation in decision making is provided in Table 1.

**Table 1:** Patient’s participation in decision making

Argument	Author
**Arguments for (based on Patient’s Autonomy)**	
Respecting patient’s right to self-determination.	(Gold, 2004).
Protection of patient-doctor relationship.	(Fallowfield et al., 2002; Tattersall et al., 2002).
Empowerment of patients’ adherence to therapy.	(Kazdaglis et al., 2010).
**Arguments against (based on beneficence)**	
Severe illness influences capacity for autonomous decision-making.	(Beste, 2005).
May lead to an abdication of doctors’ responsibility	(Gold, 2004).

## 3. Factors that Contribute to Non-Disclosure of Diagnosis

The main questions regarding the issue of truth telling are the next four:

- To what extent should the patient be informed
- Who should take the responsibility of telling the bad news (doctor, patient’s family) and to whom should the doctor disclose the diagnosis (patient or his/her relatives)
- How such disclosure should happen
- When should it happen (Shahidi, 2010).

According to findings of a number of studies there is no a clear answer for what is more stressful: being informed of a bad diagnosis or remaining with unanswered doubts. Psychological disturbances can be common, both in patients who were informed directly and in patients who guessed it through the course of their illness (Fallowfield et al., 2002; Atesci et al., 2004).

The most important contributing factors to non-disclosure are related with the three different parts that are involved in such a situation: doctor, patient and his/her relatives. Environmental and cultural factors play also an important role (Table 2).

**Table 2:** Contributing factors to non-disclosure of diagnosis

Type of factor	Factor	Author
Factors related to physicians	Not feeling comfortable taking away hope from patients	(Lin et al., 2003; Surbone, 2004)
	Difficulties to handle patients’ emotional reactions.	(Lin et al., 2003; Surbone, 2004)
	Fear that disclosure of a bad prognosis can be interpreted as a confession of inability.	(Korec & Andrasiova, 1997).
	Lack of specific education and clear guide-lines.	(Grassi et al., 2000; Tang et al., 2013)
Factors related to patients	Reluctance of a patients’ minority to be told bad news.	(Benson & Britten, 1996; Friis et al., 2003).
	Difficulties based on individual differences (sex, age, cultural and social background)	(Fallowfield et al., 2002).
Factors related to patients’ family	Effort of protecting the ill member from feelings of hopelessness.	(Noone et al., 2000; Mystakidou et al., 2004).
	Strong influence of the principle of beneficence.	(Wang et al., 2004).
Environmental factors	Lack of time.	(Shahidi et al., 2007).
	Lack of appropriate place.	
Cultural Factors	Language barriers.	(Shahidi, 2010).
	cultural differences	

Truth telling in terminally ill patients seems to be one of the most difficult tasks for doctors and other professionals of health-care system (Grassi et al., 2000; Yardley et al., 2001). Research has shown that physicians do not feel comfortable taking away the hope from patients, while there is also a strong fear of not knowing how to handle patients’ emotional reactions (Lin et al., 2003; Surbone, 2004).

In addition, informing someone of a bad prognosis, by many physicians is interpreted as a confession of inability to provide the right cure. The avoidance of such a confession maintains the image of a “good doctor” (Korec & Andrasiova, 1997).

The above difficulties are impressed in research data. The majority of the doctors indicate that they need clinical guide-lines for truth telling, while more than the half suggested the need for relevant in-service education (Grassi et al., 2000; Tang et al., 2013).

Disclosing fatal prognoses is a very hard task for healthcare professionals, (Mahtani-Chugani et al., 2010). The person delivering the “bad news” should be someone whom the patient trusts and feels most conformable with. This presupposes a certain level of communication skills and emotional capability, as a result of focused and appropriate training (Faulkner, 1998).

Except of doctor’s attribute towards truth telling, another crucial factor that determines the disclosure or non-disclosure of a diagnosis is the patient’s option. In the specific field there are contradictory data. Many studies have shown that a lot of patients with serious diseases like cancer, do not want to be told bad news (Benson & Britten, 1996; Friis et al., 2003).

On the other hand a lot of recent studies from different cultural environments suggest that most cancer patients want to know more information about their diagnosis and prognosis (Pimentel et al., 1999; Noone et al., 2000; Sapir et al., 2000; Seo et al., 2000; Arribas et al., 2004; Cox et al., 2006; Jiang et al., 2007).

Although it is not easy to extract a general assumption, the literature suggests that while most patients want to take more information, there is a substantial minority who prefers to avoid it (Kazdaglis et al., 2010).

Of course the importance of providing information for each patient is based on his/her preferences regardless of sex, age, cultural and social background and a number of specific factors that are different for each person (Fallowfield et al., 2002).

Relatives, on the other hand, usually have the tendency to exclude patients from truth telling, as a apart of an effort to protect them from feelings of hopelessness (Noone et al., 2000; Mystakidou et al., 2004).

Especially in societies where families have strong bonds, withholding is perceived as a duty of protection of the ill member. Relatives’ pressure leads physicians to inform only family members (Mitchell, 1998; Surbone, 2004).

The differences in the behavior of patients, whose majority want to know their diagnosis, and relatives, who usually prefer non-disclosure of information, show us that patients, in many cases, follow the principle of autonomy, whereas family follow the principle of beneficence, which rationalizes non-disclosure (Wang et al., 2004).

But although the family wants to protect patients, most of the times by withholding the truth, provokes the opposite result. Lack of information is perceived as a factor that can increase a patient’s uncertainty, anxiety and distress. Keeping secrets is not always effective, especially when patients finally discover their diagnosis (Tabak et al., 2012).

Another important parameter that leads to non-disclosure practices, is lack of time and the appropriate place, that characterizes the everyday clinical practice in many hospitals. Usually physicians visit too many patients every day. It is very difficult for them to find enough time to communicate with their patients in an effective way, to listen to their concerns and answer their specific questions. Especially in crowded hospitals where a private place is difficult to find (Shahidi et al., 2007).

Finally in modern multi-cultural societies, many times language barriers decrease the effectiveness of communication between doctors and patients. Especially when another person (usually a friend or a member of the family) acts as an interpreter, the information may be filtered (Shahidi, 2010).

But even if there are no language barriers, cultural differences play a crucial role in doctor-patient communication. So, an increased awareness of these differences is necessary for the achievement of the optimal health outcomes among people from different communities.

## 4. Cultural influences

The issue of truth telling to patients as far as their diagnosis, treatment options and its side effects is approached differently in various countries. It seems that there is still no global consensus, while different cultures lead to different practices (Tieying et al., 2011).

In European and North American societies direct truth telling is more culturally acceptable (De Pentheny et al., 2011). On the other hand, Asian societies prefer indirect truth telling (Mitchell, 1998; Fujimori et al., 2007). Most of these differences are basically attributed to cultural expectations and to ethnic background of both medical staff and patients (Kendall, 2006).

Besides attitudes toward truth telling are different both among different countries and among different chronic periods in the same country. Indeed, practices of western physicians have changed over time. As illustrated by two similar surveys conducted in USA in 1961, 90% of the 219 physicians examined, reported that they prefer not to disclose a cancer diagnosis, while about 20 years later 97% of the 264 physicians had a positive attitude towards revealing the diagnosis to patients with cancer (Tabak et al., 2012).

Nowadays in the USA the majority of patients are told of their diagnosis. The same happens in England, Canada and Finland. The main reason is related with current disclosure policies, which recommend that health-care professionals should provide full information. In addition, in westerns societies patients’ right to participate in decisions about their health care is safeguarded by legislation rules (Kazdaglis et al., 2010).

On the other hand in China nearly 60% of oncology physicians believed that patients who are terminally ill should not been informed of the truth. The paternalism of Chinese doctors is based in the perception that disclosure could make patients feel hopeless and helpless (Jiang et al., 2007).

Similar, in Japan only a small percentage of doctors (13%) talk openly with cancer patients about their diagnosis. Instead, they inform family members who play a crucial role in the decision whether a doctor should finally discuss with the patient (Tanida, 1994).

Paternalistic practices are also observed in Islamic cultures. Study in Iran showed that almost half of the terminally ill patients with cancer were not aware of their diagnosis. Only one out of six knew that cancer was metastatic, while none of them had had a disclosure session with their doctor (Shahidi, 2010).

In Saudi Arabia 75% of doctors discuss information with close relatives, rather than patients and in Kuwait 79% of Physicians withhold the truth after patient’s family request. Finally in Lebanon nearly half of the doctors inform patient about cancer. Besides, in this country legislation allows non-disclosure (Kazdaglis et al., 2010).

But non-disclosure is a common practice and in some Western societies where family bonds are too strong. In Greece, although most health professional believe that informing patients is very important, they avoid speaking with them about their diagnosis. In an effort to protect them from anxiety and despair feelings, usually follow family’s wish to exclude them from the information exchange (Georgaki et al., 2002). Similarly in a study in Spain nearly 70% of patients were not informed about their cancer in a terminal stage (Centeno-Cortes & Nunez-Olarte, 1994).

## 5. Can Disclosure Be Harmful?

In modern societies it is commonly acceptable as a basic ethical rule, that patients should be informed by health-care professionals about their diagnosis, prognosis and treatment. But except for the ethical guideless there is the “real world” of people, where things are sometimes more complicated and less straightforward than ethical principles.

Withholding the truth is based in the fear that bad news will drastically and negatively alter a patient’s view of their future. Moreover, it is an attempt to foster hope and to protect patients from unpleasant and difficult emotions.

It is true that in peoples mind exist two different levers of the prognosis of serious diseases like cancer. The “scientific prognosis” of the doctor and the “emotional prognosis” of the patient, which still continues carrying the historical burden of endless suffering and death (Galeazzi, 1997).

But is the avoidance of truth telling an effective strategy of protection ?

Withholding the truth affects the relationship between doctor and patient by increasing the professional’s risk losing his/her trust and confidence, which are essential to achieve better treatment outcomes (Surbone, 2004).

Moreover, according to the literature, withholding the truth can result in patients being as upset as they were lied to (Baile et al., 2000).

At the same time truth may lead to more appropriate care. Patients who are not informed may fail to get the right medical support. Sometimes in order to hide the severity of disease, intensive treatment may be substituted by other, with fewer side effects (Clayton et al., 2008).

On the contrary, realistic information, concerning different aspects of treatment may lead to more appropriate medical support and to patients’ adherence to the therapeutic schemes (Clayton et al., 2008; Kazdaglis et al., 2010).

Besides, nowadays, it is too difficult to hide the diagnosis through the whole process of treatment. Patients frequent find out the truth through media sources and Internet, which many times are not reliable. This hides both the danger for invalid information (which can provoke greater despair) and the danger of total loss of the patient’s confidence not only to the doctor but to family and relatives as well (Surbone, 2004).

## 6. Towards the Formulation of an Effective Disclosure Technique

Non-disclosure is not limited to the disrespecting of patients’ human right for self-determination. Truth telling can also contribute in the achievement of therapeutic efficacy. However an absolutistic approach is often not helpful.

What is of importance is the way that information should be given to patients. A good truth telling technique could have positive effects on their prognosis and quality of life, reducing emotional pressure and maintaining their sense of hope (Tang et al., 2013).

Truth telling does not mean that health care professionals must simple disclose all the information bluntly in one session. It is very important while sharing medical information nothing remains uncertain. Doctor has to be aware of the way that truth is affecting each patient and respond in an empathetic and attentive manner (Beste, 2005).

Sometimes too much information can be overwhelming. Doctors during the process of disclosure must take into account patients’ characteristics, personal needs, preferences and beliefs (Fallowfield et al., 2002).

Of course disclosure policy is affected a lot by cultural context, which determines the boundaries in communication objectives. In Asian and South-east Europe countries physicians should be more willing to answer patients’ questions, taking into account cultural issues, which make difficult the total revealing of diagnosis. In USA and Northern countries, it is easier to adopt a more direct attitude. Avoiding absolutistic approaches, health care professionals can search for a consensus between the two extremes of total disclosure and non-disclosure (Kazdaglis et al., 2010).

An appropriate disclosing technique can give patients a sense of control, increasing their hopefulness. Being able to confide in doctors their fear, they may improve their ability to cope with their suffering (Beste, 2005).

Consequently, the dilemma in clinical practice does not limit to disclosure or non-disclosure. The way that information should be given is of great importance. Health professionals must tailor their approach to individual needs, since each one of the patients consists of a different occasion.

The respect for patient’s autonomy imposes presenting all treatment options, explaining risks in terms that he/she understands and ensuring that after understanding he/she will agree to all necessary procedures.

Of course, since each person is different, it is very difficult to find a strategy for fostering hope when discussing prognosis, which could be appropriate for all. However the following issues could be very helpful:

- Determine individual preferences for information.
- Be open to discussions about prognosis, without being blunt or giving too much information and details, that often bring more confusion to the patient.
- Give emphasis to what is available in the field of treatment, as well as in the other available support services throughout the illness trajectory.
- Be more specific in the identification of the available treatments and applications for controlling pain and other symptoms and of the areas where control can be fostered (like the advance care planning).
- Recognize the forms of hope that are possible even in the face of a serious or terminal illness (Clayton et al., 2008).

## 7. Conclusion

Balancing hope with honesty is one of the most important skills of the art of medicine. Although bad news is always bad, however it is said, the way of conveying has a crucial effect, not only to the patient, but also on the care-giver (Narayanan et al., 2010).

The literature suggests that issues surrounding hope in the context of breaking bad news are too complex. However, some key communication techniques may facilitate the flow of information in breaking bad news interview.

Beginning from the setting up stage, professionals must arrange for some privacy and manage time constrains and interruptions. Significant others (relatives) can be involved but only according to patient’s choice (Baile et al., 2000).

Assessing patient’s perception about illness is also important. The axiom “before you tell, ask” can be proved very useful to this direction. Open-ended questions can create an accurate picture of how patient perceives his/her medical situation (Lubinsky, 1994).

Meanwhile, some simple guidelines can be proved very useful in giving medical facts during physician-patient dialogue. Starting at the level of patient’s comprehension and vocabulary, avoiding using technical words, giving information in small chunks, checking the patient’s understanding and avoiding excessive bluntness, may help the whole procedure (Maynard, 1996).

One of the most difficult challenges of breaking bad news is empathic responding to patient’s emotions. Empathic response consists of four steps: observing for all emotions, identifying them by name them to oneself, identifying the reason for it and letting the patient know that we have understood him/her, through a statement that can reflect it (Ptacek & Eberhardt, 1996).

At the end of breaking bad news session the physician must always summarize the concerns expressed by the patient, highlighting the main points of their transaction. This can give to the patient a higher level of emotional holding, especially when the doctor offers availability and encourages the patient to call for any reasons (Narayanan et al., 2010).

The above presupposes a certain level of communication skills and emotional capability, as a result of a focused and appropriate training. Clear guidelines must be integrated in the academic education of health care professionals, as also in their continuous in-service education. Meanwhile, further research about clinicians and patients attitudes towards revealing the truth during a serious illness and the influence of social and cultural context above them is definitely needed.
